# miR-429 regulates the transition between Hypoxia-Inducible Factor (HIF)1A and HIF3A expression in human endothelial cells

**DOI:** 10.1038/srep22775

**Published:** 2016-03-08

**Authors:** Anna Janaszak-Jasiecka, Sylwia Bartoszewska, Kinga Kochan, Arkadiusz Piotrowski, Leszek Kalinowski, Wojciech Kamysz, Renata J. Ochocka, Rafał Bartoszewski, James F. Collawn

**Affiliations:** 1 Department of Biology and Pharmaceutical Botany, Medical University of Gdansk, Gdansk, Poland; 2 Department of Inorganic Chemistry, Medical University of Gdansk, Gdansk, Poland; 3 Department of Medical Laboratory Diagnostics, Medical University of Gdansk, Gdansk, Poland; 4 Department of Cell, Developmental and Integrative Biology, University of Alabama at Birmingham, Birmingham, USA

## Abstract

Hypoxia-inducible factors (HIF) are heterodimeric transcription factors that allow cells to adapt and survive during hypoxia. Regulation of *HIF1A* and *HIF2A* mRNA is well characterized, whereas *HIF3A* mRNA regulation and function are less clear. Using RNA-Seq analysis of primary human umbilical vein endothelial cells, we found two isoforms of *HIF3A* were expressed, *HIF3A2* and *HIF3A3*. Comparing *HIF3A* expression profiles to *HIF1A* mRNA during 48 hours of hypoxia revealed that *HIF1A* message peaked at 4 hours, whereas *HIF3A* expression increased while *HIF1A* was decreasing. Given that *HIF1A* mRNA is regulated by miR-429, we tested miR-429 effects on both *HIF3A* isoforms and found that they too were regulated by miR-429. Analysis of a HIF-3 target, DNA-damage-inducible transcript 4, a key survival gene, indicated that *DDIT4* mRNA is induced by HIF-3 and negatively regulated by miR-429 through miR-429’s actions on *HIF3A* message. This provides a compelling model for how hypoxia-induced miR-429 regulates the switch between HIF-1 adaptive responses to HIF-3 survival responses by rapidly decreasing *HIF1A* levels while simultaneously slowing the progression of *HIF3A* expression until the miR-429 levels drop below normoxic levels. Since HIF-1 drives *HIF3A* and miR-429 expression, this establishes a regulatory network in which miR-429 plays a pivotal role.

Ischemia-reperfusion injury results in tissue damage in organs such as the heart where hypoxia and reperfusion lead to fibrotic remodeling and cardiomyocyte apoptosis^1^. During the initial stages of hypoxia, a cellular adaptive response is induced that minimizes cell death through activation of three transcription factors called hypoxia-inducible factors (HIFs) that respond to low tissue oxygen levels and stimulate the expression of a number of genes that promote angiogenesis, energy metabolism, and cell survival^2^,^3^. The genes activated by HIF-1 enhance oxygen delivery to the tissues and/or promote cellular metabolic adaption to the reduced oxygen levels^3^. HIF-1, HIF-2 and HIF-3 are tightly regulated through changes in oxygen tension^4^,^5^. They consist of stable, constitutively expressed β subunits that associate with the oxygen destabilized α subunits^6^. During normoxia, postranslational hydroxylations of alpha subunits target them for polyubiquitination and degradation^7^,^8^. During hypoxia, however, the alpha subunits are stable and translocate to the nucleus to dimerize with the β subunits to create transcriptionally active HIF-1, HIF-2 and HIF-3 complexes^5^.

HIF-1 and HIF-2 are widely accepted as major mediators of the hypoxic response^9^. The HIF-1 and HIF-2 function as transcriptional activators and have both unique and overlapping target genes. HIF-1 governs initial adaptation to hypoxia, whereas HIF-2 expression begins after more prolonged oxygen depletion^9^,^10^. Understanding the exact roles of HIF-3 are complicated by the fact that there are alternatively spliced variants of *HIF3A* and *HIF3A* utilization of different promoters results in at least four different mRNA variants that code for six or more isoforms^11^,^12^,^13^. Furthermore, overexpression of the HIF-3α isoforms, suppresses HIF-1 and HIF-2 activity in cell culture^14^,^15^. Recent studies, however, indicate that human HIF-3 isoforms have transcriptional functions that partially overlap with that of HIF-1, indicating that HIF-3 is more than just a dominant-negative form that suppresses HIF-1 and HIF-2 function^16^,^17^.

Previous studies have demonstrated that a number of microRNAs (miRNAs) are induced during hypoxia and play critical roles in angiogenesis^18^,^19^,^20^. Some of these miRNAs, miR-210-3p and miR-485-5p, have been shown to regulate *HIF-3A* mRNA in soft tissue sarcoma cells^21^. Our previous studies demonstrated that miR-429 is induced during hypoxia in primary human umbilical vein endothelial cells (HUVECs) and regulates HIF-1 mRNA levels during the early stages of hypoxia^10^.

Here we examined whether miR-429 played any role in the later stages of hypoxia, and interestingly, found an inverse relationship between *HIF3A* mRNA levels versus miR-429 and *HIF1A* expression. We analyzed this in more detail and found that miR-429 binds directly to *HIF3A2* and *HIF3A3* 3′UTRs and regulates their expression. We also demonstrate that miR-429 regulates a transcriptional target of HIF-3, DNA-damage-inducible transcript 4 (*DDIT4*), a pro-survival protein that inhibits the mechanistic target of rapamycin (mTOR) complex 1 (mTORC1). Our studies suggest that miR-429, which is induced early in the hypoxia time-course, regulates the transitional switch between HIF-1 and HIF-3 responses in HUVECs during prolonged hypoxia by first attenuating HIF-1 responses during the early stages of hypoxia by destabilizing *HIF1A* message and then delaying the onset of *HIF3A* message stability in the later stages. Given that HIF-1 drives the expression of *HIF3A* and miR-429, this establishes a regulatory network in which miR-429 plays a critical role.

## Results

### *HIF3A2* and *HIF3A3* mRNAs are expressed in human endothelial cells

**Figure 1:**
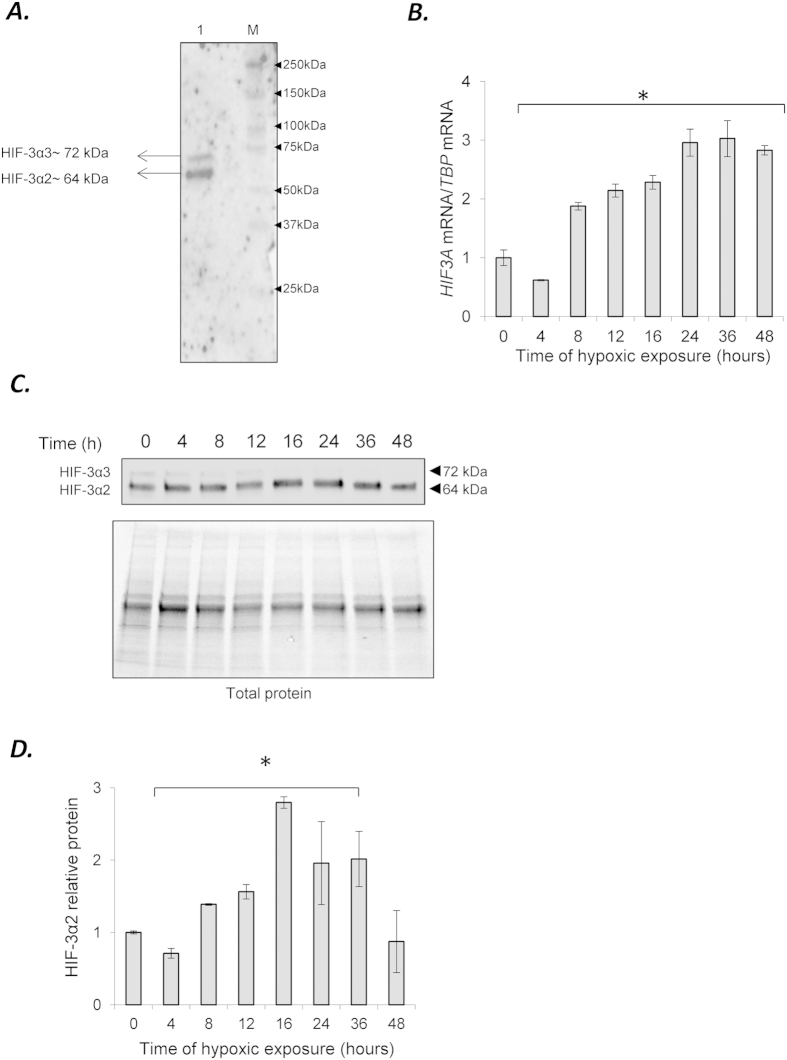
Expression of HIF3α isoforms in HUVECs. **(A)** The anti-HIF-3α (Sigma-Aldrich, AV39936) antibody allows detection of HIF-3α2 (~72 kDa) and when overexposed, HIF-3α1 (~72 kDa). The HUVECs cell lysate is shown in lane 1 (5 μq loaded), and the molecular weight markers (M) are shown in lane 2 (Precision plus Kaleidoscope, BioRad #161-0375). **(B)** Hypoxia induces dynamic changes in the mRNA expression profiles of the *HIF3A2–3 variants* in HUVECs. The mRNA levels were monitored in qRT-PCR experiments. The results from 3 independent experiments (n = 12) are plotted normalized to TBP mRNA levels and expressed as a fold-change over the normoxic control. Error bars represent standard deviations (^*^P < 0.05). **(C)** Hypoxia induces dynamic changes of protein levels of HIF-3α2 and **(D)** the bar graphs show the relative protein amounts at each time point. The protein levels of were detected with SDS-PAGE and Western Blot and related to total protein levels. 2 individual samples (4 μg of total protein per lane) were tested for each time point and the experiments were repeated twice. The protein levels (bar graphs) are normalized to the normoxic control. Significant changes (p < 0.05) are marked with an “^*^”.

Given the complexity of the *HIF3A* locus, we used next generation sequencing analysis followed by qRT-PCR validation to determine which *HIF3A* isoforms were expressed in HUVECs. The data indicates that *HIF3A* is expressed at low levels, and only isoforms *HIF3A2* and *HIF3A3* were detected (Supplemental Figure 1). These two HIF3A isoforms are annotated in the RefSeq database (ncbi.nlm.nih.gov/refseq/: NM_022462.4 and NM_152795.3, respectively). The protein expression supported the mRNA analyses and demonstrated that HIF-3α2 protein is expressed at a higher level than HIF-3α3 (64 kDa form versus the 72 kDa form, respectively (Fig. 1A). In our protein analysis, furthermore, it was difficult to the detect HIF3α3 isoform unless the blots were overexposed as shown in (Fig. 1A, and therefore we focused our subsequent analyses on HIF-3α2. To follow the expression of *HIF3A* message levels during hypoxia, the HUVECs were treated from 0 to 48 h in 1% oxygen and the mRNA levels were measured (Fig. 1B). The results indicate that *HIF3A* mRNA, a measure of both *HIF3A2* and *HIF3A3*, is reduced below normoxic levels after 4 hours of hypoxia and then gradually increases ~3-fold by 24 hours and remains elevated for 48 hours. To evaluate the consequences of hypoxia on the protein expression levels of the HIF-3α, we monitored expression by western blot analysis. The HIF-3α2 proteins levels mirrored the mRNA levels with the exception of the 48 hours time point that appeared similar to the normoxic start conditions (Fig. 1C,D). Interestingly, the HIF-3α2 protein levels do not begin to rise above normoxic levels until 8 hours of hypoxia. At 4 hours, the HIF-3α2 protein levels drop significantly and follow the mRNA changes, although the mRNA levels appear to rebound much faster than the corresponding protein changes.

**Figure 2:**
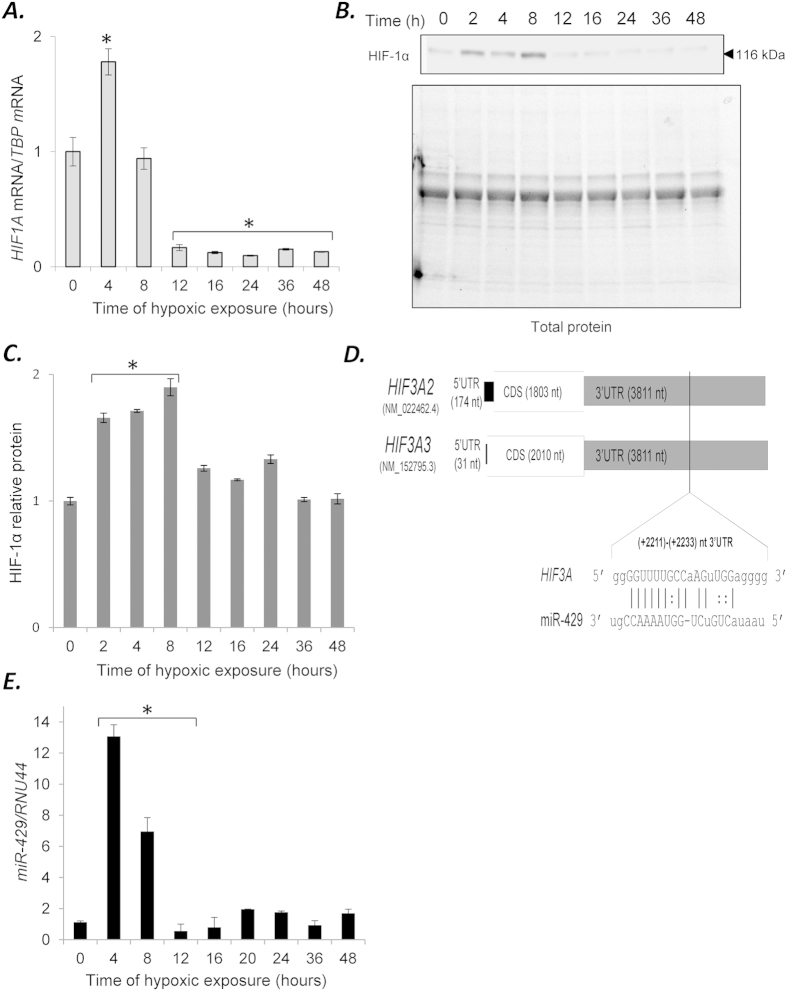
**(A)** Hypoxia induces dynamic changes in the mRNA and protein expression profile of the *HIF1A* in HUVECs. The mRNA levels were monitored in qRT-PCR experiments. The results from 3 independent experiments (n = 12) are plotted normalized to TBP mRNA levels and expressed as a fold-change over the normoxic control. Error bars represent standard deviations. **(B)** Hypoxia induces dynamic changes of protein levels of HIF-1α and **(C)** the bar graphs show the relative protein amounts at each time point. The protein levels of were detected with SDS-PAGE and Western Blot and related to total protein levels. 2 individual samples (4 μg of total protein per lane) were tested for each time point and the experiments were repeated twice. The protein levels (bar graphs) are normalized to the normoxic control. Significant changes (p < 0.05) are marked with an “^*^”. **(D)** The predicted target site of miR-429 in *HIF3A2-3.* The localization of putative miR-429 sequence is shown aligned with the 3′UTR of *HIF3A* isoforms. The numbering is based on the human NCBI genomic sequence (RefSeq). **(E)** Hypoxia-induced changes in the expression profile of miR-429 in HUVECs. The miRNA level was monitored in quantitative real-time PCR experiments. The results from 3 independent experiments (n = 18) are plotted normalized to *RNU44* and expressed as a fold change over the normoxic control (^*^P < 0.05).

Given that HIF-3α is regulated by HIF-1 but not HIF-2^13^, we next examined the mRNA levels of *HIF1A* under these same conditions (Fig. 2A). The results indicate that *HIF1A* mRNA was rapidly elevated at 4 hours, but decreases dramatically below the normoxic levels after 12 hours of hypoxia. The protein profile of HIF-1α correlated with the mRNA analysis with the protein elevated from 2 to 8 hours (Fig. 2B,C). The *HIF1A* profile negatively correlated with the *HIF3A* mRNA, emphasizing the temporal segregation between the *HIF1A* and *HIF3A* expression patterns in HUVECs (Supplemental Figure 2).

In our previous studies, we found that miR-429 was induced by HIF-1, and that *HIF1A* mRNA was regulated by miR-429 in a negative feedback loop^10^. In examining the 3′UTR of the *HIF3A* isoforms using RNAhybrid software^22^, we identified a potential miR-429 targeting sequence in the *HIF3A* 3′UTRs (Fig. 2D). To establish whether there was a correlation between miR-429 and the *HIF3A* mRNA expression during hypoxia, we examined the same HUVEC samples used for the mRNA analyses and monitored for miR-429 expression. The results shown in Fig. 2E demonstrate that miR-429 was elevated ~13-fold at 4 hours, and then rapidly declined below normoxic levels by 12 hours and remained low for the duration of the time course. Interestingly, the miR-429 profile negatively correlated with the *HIF3A* mRNA, suggesting that miR-429 expression could be responsible for initial reduction of *HIF3A* levels (Supplemental Figure 2).

### miR-429 regulates *HIF3A* mRNA steady-state levels

**Figure 3:**
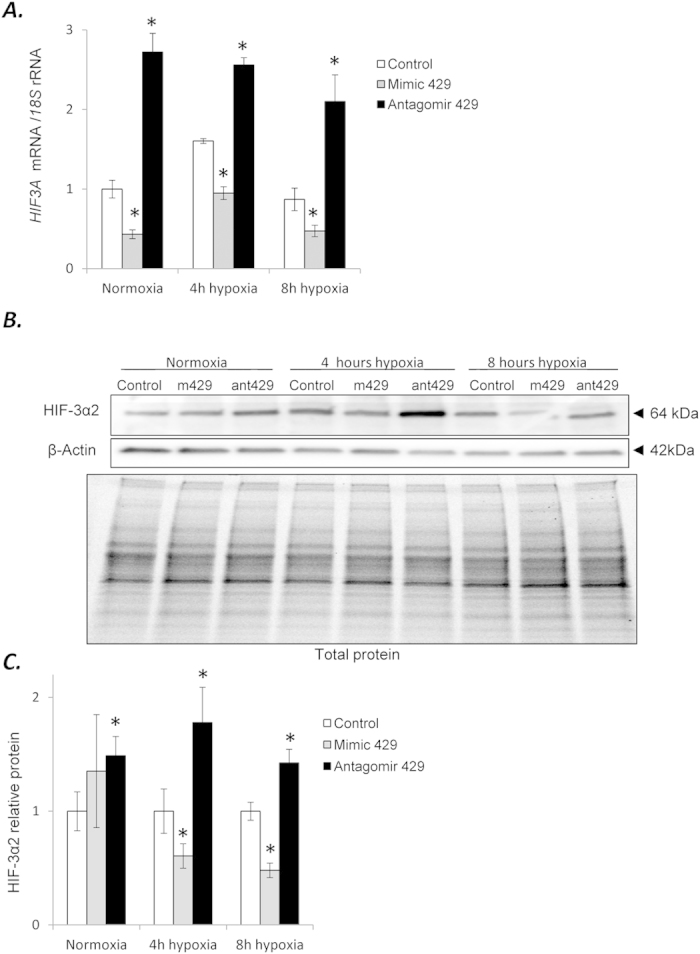
(**A**) miR-429 alters the expression of *HIF3A*. HUVECs were transfected with miR-429 mimic or antagomir, and the mRNA levels were monitored in normoxic conditions and after 4 h and 8 h of exposure to hypoxia. *HIF3A* mRNA levels from 3 independent experiments (n = 12) are plotted normalized to *18S* rRNA levels and expressed as a fold change over the transfection control. Significant changes (p < 0.05) are marked with an “^*^”. (**B**) The corresponding changes of HIF-3α2 protein levels of were detected with SDS-PAGE and Western Blot and normalized to the β-actin and total protein levels. **(C)** The bar graphs show the relative protein amounts at each condition normalized to the transfection control. **(D)** 2 individual samples (4 μg of total protein per lane) were tested for each treatment and the experiments were repeated twice. Mimic 429 (m429) and antagomir 429 (ant429) are shown for each condition.

We next tested whether miR-429 affected *HIF3A* mRNA and HIF-3α2 protein levels by transfecting the HUVECs with miR-429 mimics or antagomirs and followed their effects on *HIF3A* mRNA expression under both normoxic and hypoxic conditions. We first established that the transfection efficiencies promoted appropriate effects on the miR-429 levels in the HUVECs (Supplemental Figure 3). Examining the functional effects indicated that inhibition of miR-429 with antagomir 429 resulted in the accumulation of *HIF3A* mRNA, whereas miR-429 mimic expression reduced *HIF3A* mRNA levels (Fig. 3A). Furthermore under these same conditions, the HIF-3α2 protein levels followed the same pattern as the mRNA except for the miR-429 mimic transfection during normoxic conditions (Fig. 3B,C). Given the instability of the HIF3α-chains during normoxic conditions, however, this could explain the limited correlation here.

**Figure 4:**
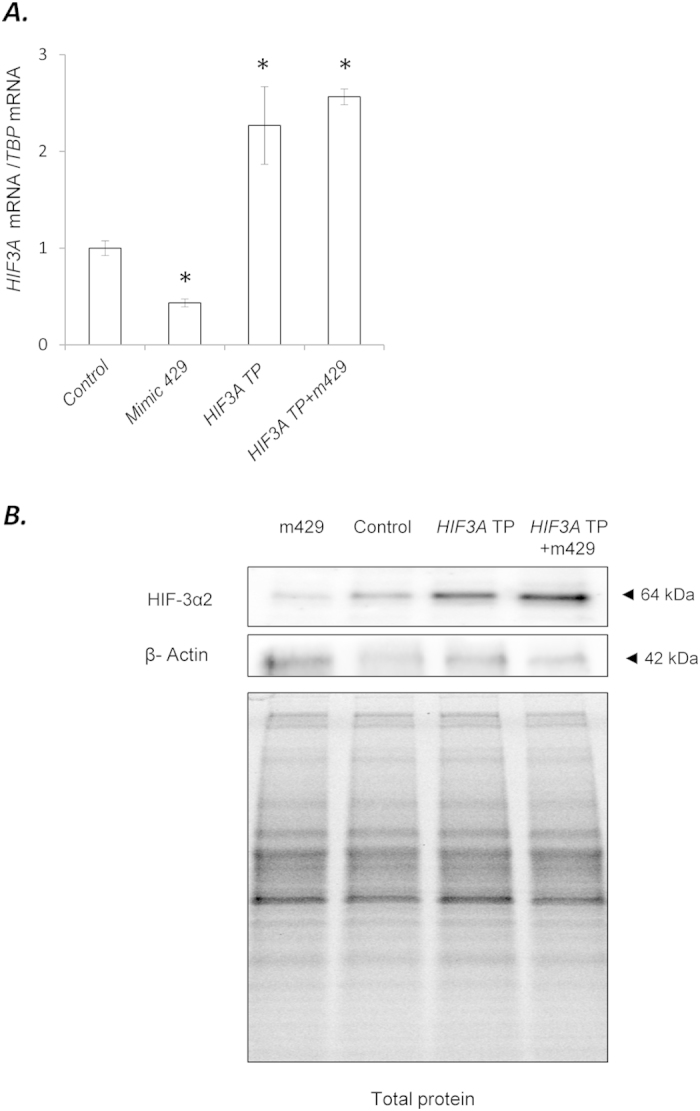
miR-429 binds to predicted target sequence in the HIF3A1-3 3′UTR. **(A)** HUVECs were transfected with *HIF3A* target sequence-specific target protector and/or the miR-429 analog (Mimic 429). Two days after transfection, the cells were placed in hypoxia for 4 h and the *HIF3A* mRNA levels were monitored in qRT-PCR experiments. The *HIF3A* levels results from 3 independent experiments (n = 12) are plotted normalized to *TBP* mRNA levels and expressed as a fold change over the transfection control. Significant changes (p < 0.05) are marked with an “^*^”. The corresponding changes of HIF-3α2 protein levels were detected with SDS-PAGE and Western Blot and normalized to the β-actin levels and total protein levels. **(B)** 2 individual samples (3 μg of total protein per lane) were tested for each treatment and the experiments were repeated twice.

In order to test if miR-429 directly interacted with the predicted *HIF3A* target sequence that we identified, we used a specific target protector to inhibit the interaction between miR-429 and the *HIF3A* mRNA. Target protectors are modified RNAs complementary to miRNA target sequences that bind to the specific mRNA 3′UTR target sequence and block miRNA binding, thus preventing the formation of the predicted miRNA-mRNA complex shown in Fig. 2B^23^. As shown in Fig. 4A, the *HIF3A* target protector (TP) blocked the miR-429 mimic effects on the *HIF3A* mRNA levels. The corresponding protein changes in these same samples showed that the *HIF3A* TP increased the HIF-3α2 levels, blocked the effect of mimic 429, and that mimic 429 alone dramatically decreased HIF-3α2 protein levels below normoxic control levels (Fig. 4B). The results suggested that the predicted target sequence identified was correct, and that under hypoxic conditions miR-429 regulates the levels of the *HIF3A* mRNA and protein levels.

### miR-429 affects the *HIF3A* target *DDIT4* mRNA levels indirectly

**Figure 5:**
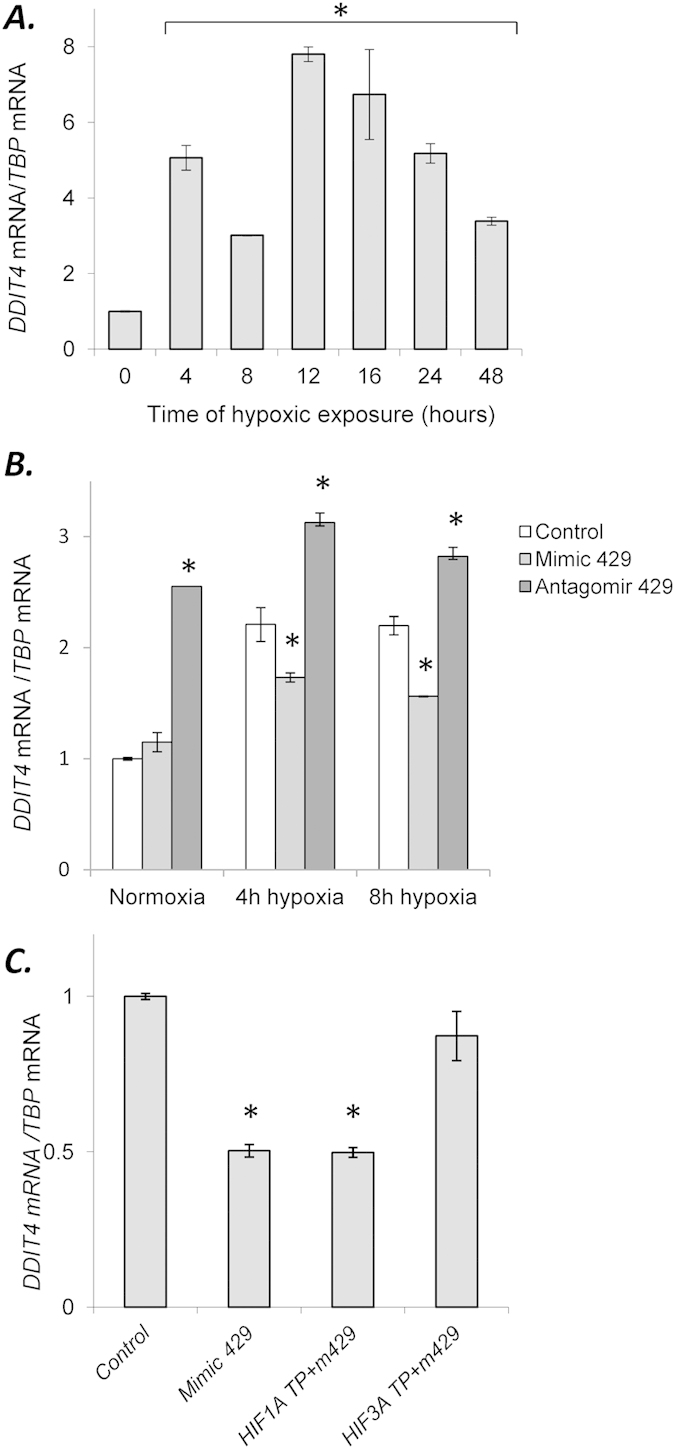
Hypoxia induces dynamic changes in expression profiles of *DDIT4* **(A)** in HUVECs. The results from 3 independent experiments (n = 18) are plotted normalized to *TBP* mRNA levels and expressed as a fold-change over the normoxic control. Error bars represent standard deviations. miR-429 mimic and antagomir modulate *DDIT4* expression. **(B)** HUVECs were transfected with miR-429 mimic or antagomir and the mRNA levels were monitored in qRT-PCR experiments in normoxic conditions and after 4 and 8 hours of hypoxia. The mRNA levels from 3 independent experiments (n = 12) were normalized *TBP* mRNA levels and expressed as a fold change over the transfection control. miR-429 indirectly modulates the expression of *DDIT4* mRNA levels during hypoxia through its actions on *HIF3A* mRNA. **(C)** mRNA levels were monitored in qRT-PCR experiments following transfection of HUVECs with *HIF3A* TP and *HIF1A* TP together with miR-429 analog (Mimic 429). The mRNA levels results from 3 independent experiments (n = 12) are normalized to *TBP m*RNA levels and expressed as a fold-change over the transfection control. Significant changes (p < 0.05) are marked with an “^*^”.

In order to test if miR-429 has a functional influence on HIF-3α targets, we analyzed the hypoxia-driven expression of gene previously identified as a HIF-3α transcriptional target in HEK293 cells, *DDIT4*^16^. *DDIT4* regulates cell growth and survival via inhibition of the activity of mTORC1, and therefore the induction of this gene during hypoxia promotes cell survival^24^,^25^,^26^. First, we followed the hypoxia time-course for *DDIT4* mRNA as described before and found that its expression was biphasic with a first peak at 4 hours (Fig. 5A). To test if miR-429 affected *DDIT4* mRNA levels during normoxia or hypoxia, the cells were transfected with mimic 429 and antagomir 429. The results shown in Fig. 5B indicated that *DDIT4* mRNA levels were affected significantly with both mimic and antagomir at 4 and 8 hours of hypoxia. During normoxic conditions, the antagomir, but not the mimic, had a significant effect.

Given that the mimic did not affect the *DDIT4* mRNA during normoxia, this suggested that the miR-429 effect on *DDIT4* could be indirect. To test for this, we transfected the HUVECs with *HIF3A* and *HIF1A* target protectors and examined their ability to block the mimic 429 responses after 4 hours of hypoxia. The results shown in Fig. 5C show that the *HIF3A* target protector, but not *HIF1A* target protector, blocked the mimic 429 decrease in *DDIT4* mRNA levels, indicating that miR-429 was inhibiting *DDIT4* expression through its actions on *HIF3A* mRNA, and therefore miR-429 effects on *DDIT4* mRNA expression were indirect.

To confirm that HIF-1 enhances the expression of *HIF3A* and miR-429, we chemically enhanced HIF-1α stability and followed the levels of *HIF3A* mRNA and miR-429 during this treatment (Supplemental Figure 4). The HUVECs were treated with chemical hypoxia mimetics 100 μM desferoxamine (DFO) or 200 μM CoCl_2_ that inhibit proline hydroxylase activity during normoxia. This treatment stabilizes HIF-1α protein and leads to HIF-1 transcriptional activity^27^,^28^. The results show that the DFO treatment had the greatest effect in increasing *HIF3A* and miR-429 expression (Supplemental Figure 4, white bars). To confirm that the effects were due to HIF-1 activity, a functional inhibitor of HIF-1, topotecan (500 μM TPT)^29^, was used in combination with the two different hypoxia-mimetics and the results show that this inhibited the hypoxia-mimetic treatment (Supplemental Figure 4, black bars), confirming that HIF-1 was enhancing both *HIF3A* and miR-429 expression.

### miR-429 regulates the levels of *HIF3A* and *HIF1A* during hypoxia

**Figure 6:**
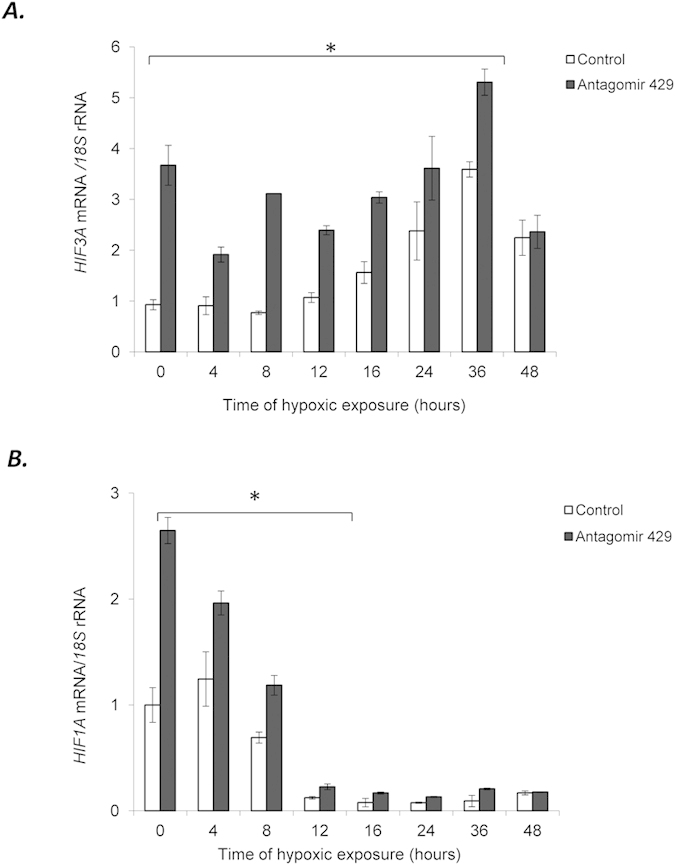
miR-429 regulates the levels of *HIF3A* and *HIF1A* mRNAs during normoxic and hypoxic conditions in HUVECs. HUVECs were transfected with miR-429 antagomir and the mRNA levels were monitored over the 48 h time course for *HIF3A* **(A)** and *HIF1A* **(B)**. The mRNA levels were monitored in qRT-PCR experiments. The results from 2 independent experiments (n = 6) are plotted normalized to *18S* rRNA levels and expressed as a fold-change over the normoxic transfection control. Error bars represent standard deviation. Significant changes (p < 0.05) are marked with an “^*^”.

To establish the physiological effects of miR-429 during the hypoxia time course in HUVECs and its corresponding effects on *HIF3A* and *HIF1A* expression, the cells were transfected with 429 antagomir and then followed over a 48-hour hypoxia time course. The results demonstrate that miR-429 is normally active up to 36 hours since the antagomir dramatically increased the levels *HIF3A* message out to that time point (Fig. 6A). For *HIF1A* message, the antagomir only had pronounced effects during the early stages since by 8–12 hours; the *HIF1A* message was back to normoxic levels (~8 hours) and substantially below normoxic levels from 16 to 48 hours (Fig. 6B). These results indicate that miR-429 regulates both the early and late stages of the hypoxic response in HUVECs by regulating the mRNA levels of both *HIF1A* and *HIF3A*.

## Discussion

HIF-1 is the master regulator for the expression of genes in response to hypoxia in most mammalian cells and its function and regulation are well established^3^. Much less is known about HIF-3 and it has been suggested that its expression is much more restricted^7^. Although HIF-1 and HIF-2 have 48% sequence identity, HIF-3 is very distinct from the other two hypoxia-induced transcription factors^7^. Previous studies in A549 cells (a human alveolar cell carcinoma cell line) indicated that *HIF1A* mRNA levels did not increase during hypoxia or when treated with 250 mM CoCl_2_, whereas *HIF3A* message increased at 4 hours and was maximal at 8 hours^17^. The differences between their studies and ours could be explained by differences in alveolar and endothelial cell regulation or because of the differences between transformed and primary cells. Our time course was much more extended, 48 hours versus 16, and an interesting feature in our studies was the clear demarcation between the *HIF1A* and *HIF3A* mRNA expression patterns and the fact that the *HIF3A* message was still elevated at 36 hours. We also demonstrate that a microRNA, miR-429, plays a key regulatory role in mediating this demarcation.

In previous studies, we demonstrated that HIF-1 drives the expression of miR-429^10^. Here we demonstrate that miR-429 attenuates the expression of *HIF3A* during the early stages of hypoxia. During normoxic conditions, the protein expression levels of HIF-1α and HIF-3α are regulated through postranslational hydroxylations of the alpha subunits that lead to subsequent polyubiquitination and degradation^8^. During hypoxia, the HIF-1α and HIF-3α proteins are stable. In the initial stages of hypoxia, the adaptive phase is driven by HIF-1. HIF-1 accumulation leads to induction of miR-429 expression that subsequently targets *HIF1A* mRNA in a negative feedback loop^10^. miR-429 also provides the switch between HIF-1 and HIF-2 activities since it reduces *HIF1A* message before *HIF2A* expression is induced at 12 h^10^. *HIF2A* does not have a miR-429 targeting sequence in its 3′UTR and therefore its message is not predicted to be affected by miR-429 expression, suggesting that miR-429 effects are limited to *HIF1A* and *HIF3A* mRNA.

*HIF1A* levels go rapidly up during hypoxia and then decline rapidly based on the actions of miR-429^10^. Here, we demonstrate that miR-429 also regulates *HIF3A* levels, and miR-429 attenuates the expression until the later time points, thus regulating the transition from *HIF1A* to *HIF3A* and presumably the temporal expression of their target genes in HUVECs. During hypoxia, *HIF1A* is up at 4 hours, *HIF2A* is up by 12 hours^10^, and *HIF3A* is initially down at 4 hours and then gradually rises from 12 to 24 h at a time when the miR-429 levels are low. The role for miR-429 is best demonstrated when the cells are transfected with 429 antagomir. Under these conditions, *HIF3A* levels are elevated from the start, even during normoxic conditions. Clearly, part of this elevation is mediated by the increases in *HIF1A* expression (Fig. 6B); however, the *HIF3A* target protector studies demonstrate that this is a direct effect on *HIF3A* as well (Fig. 4A).

As shown in Supplemental Figure 1, the *HIF3A* mRNA levels are very low (up to 50-fold lower than that of *HIF1A*). Hence, the transcriptional increase of miR-429 levels during 4 h time point will not affect the *HIF3A* mRNA levels since these are already very low, in part due to the miR-429 steady-state normoxic levels. At later time points, the decrease in miR-429 levels in combination with the HIF-1-dependent induction of *HIF3A* expression and the long half-life of *HIF3A* mRNA^17^, drives the equilibrium towards *HIF3A* mRNA accumulation. This statement is supported by the miR-429 inhibitor data illustrating that HIF3A expression increases during both normoxia and hypoxia (Fig. 3). More importantly, *HIF3A* mRNA and miR-429 are only part of mechanism that controls HIF^17^ expression during hypoxia. The changes in HIF-3α expression result from the combined effects of HIF-1, the hypoxia impaired activity of PHDs and FIH, miRNAs, and potentially other factors. Furthermore, miR-429 affects numerous other hypoxia-related targets including HIF-1^10^. Therefore, the correlation between the *HIF3A* and miR-429 levels during the hypoxia time course, although is supported by miRNA analog studies, results from a number of multiple co-dependent factors.

The transition from *HIF1A* to *HIF2A* to *HIF3A* may be critical for cell survival during prolonged periods of hypoxia. This successful transition is further supported by the early *HIF1A* induction that drives the expression of *HIF3A*. HIF-1 and HIF-3 are both strongly connected by miR-429 regulation as well as by the fact that HIF-1, but not HIF-2, drives the expression of *HIF3A*^13^. The functional relationship between HIF-1 and HIF-3 has two main consequences. First, it allows for the switch between HIF-1 and HIF-3 signaling in the HUVECs. And secondly, it prevents the continuous HIF-1 transcriptional activity through the reported dominant negative function of HIF-3α^15^. In examining the functional consequences of HIF-3α expression, we tested a potential transcriptional target, *DDIT4*. Our studies support the view that HIF-3 induces the pro-survival gene *DDIT4* and this induction is modulated by miR-429 through its direct interactions with *HIF3A* message. However, the physiological consequences of miR-429 interaction with *HIF3A* on *DDIT4* expression require further studies.

**Figure 7:**
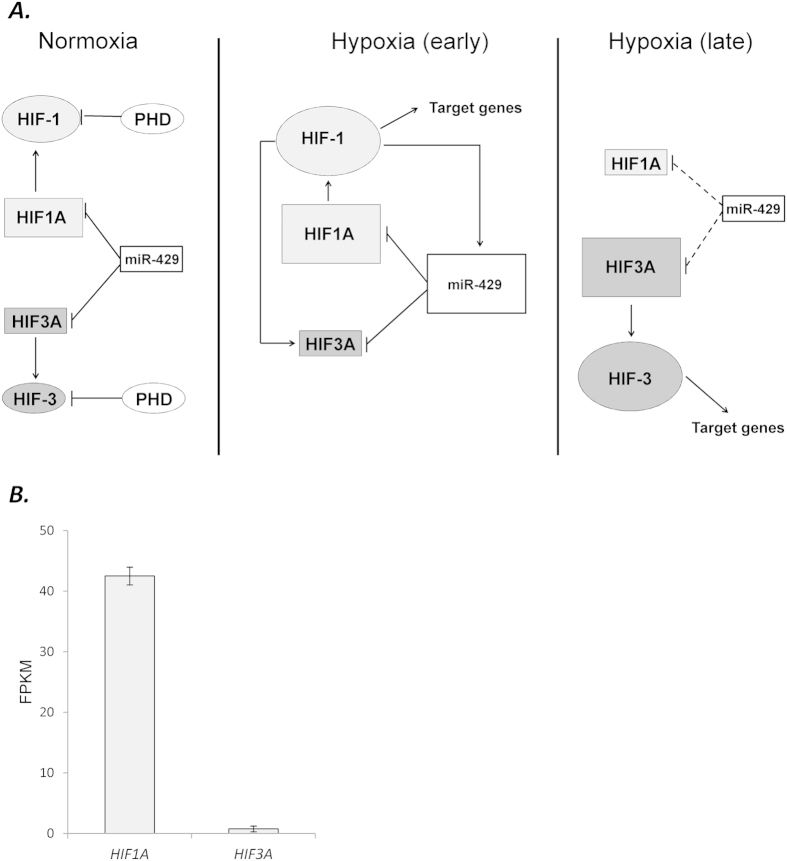
**(A)** Model of the miR-429 regulatory effects on the HIF-1/HIF-3 transition in HUVECs during hypoxia. During normoxia, miR-429 controls steady-state levels of *HIF1A* and *HIF3A* mRNA and keeps the levels low. Protein levels remain low through posttranslational modifications and subsequent degradation of these transcription factors when the oxygen levels are normal. During the early stages of hypoxia, *HIF1A* message and protein are dramatically elevated, while the *HIF3A* variants and protein remain low. HIF-1α drives a number of pro-survival and angiogenic target genes and also promotes miR-429 and *HIF3A* up regulation. miR-429 decreases the steady-state levels of both *HIF1A* and *HIF3A* mRNA. During prolonged hypoxia, HIF-1α levels and miR-429 are low, and *HIF3A* mRNA and HIF-3α protein accumulate over time, leading to the induction of HIF-3 pro-survival gene expression. **(B)** The HIF1A and HIF3A relative expression levels (Fragments Per Kilobase of transcript per Million mapped reads) in HUVECs, analysis made base on 2 NGS analysis of 2 independent samples of normoxic HUVEC cells passage 3. Error bars represent SEM.

In summary, our studies establish a model for how a microRNA that is induced during hypoxia can regulate the HIF-1/HIF-3 transition in HUVECs during hypoxia. In this model, normal oxygen levels in the HUVECs and moderate levels of miR-429 maintain low levels of *HIF1A* and *HIF3A* mRNA (Fig. 7). HIF-1α and HIF-3α protein levels remain low through posttranslational degradation of these transcription factors when the oxygen levels are normal (reviewed in)^30^. During the early stages of hypoxia, *HIF1A* message and HIF-1α protein are dramatically elevated, while the *HIF3A* mRNA isoforms and protein remain low. HIF-1 drives a number of pro-survival and angiogenic target genes and also promotes miR-429 and *HIF3A* up-regulation. miR-429 inhibits the message stabilities of both *HIF1A* and *HIF3A*. During prolonged hypoxia, HIF-1α levels and miR-429 are lower, and *HIF3A* message and HIF-3α protein accumulate over time, leading to the induction of HIF-3 pro-survival gene expression. The model shown here provides a basis for how a miRNA, which is induced early in the hypoxia time-course, regulates the transitional switch between HIF-1 and HIF-3 responses in HUVECs during prolonged hypoxia and thus regulates the transition between the induction of the early response genes to the later stages of hypoxia where other targets are induced to provide the cell with a mechanism for surviving chronic stress conditions.

## Methods

### Cell lines and culture conditions

HUVECs were obtained from ATCC and maintained until passage six in EGM-2 Bulletkit medium (Lonza). Cells were split either into 6-well plates or 10 cm dishes and allowed to grow to 70–80% confluence prior to the start of the experiments.

### Induction of hypoxia

Hypoxia was induced in a CO_2_/O_2_ incubator for hypoxia research (Binder CB160 with O_2_ control, trigas model) and oxygen levels additionally monitored with an independent oxygen meter (PCS GOX100). Briefly, cells were cultured in 10 cm dishes at 1% O_2_ for the time periods specified. Control cells were maintained in normoxic conditions in the same incubator and harvested at the specified times.

### Isolation of RNA and microRNA

Total RNA containing the microRNA fraction was isolated using miRNeasy kit (Qiagen). RNA concentrations were calculated based on the absorbance at 260 nm. RNA samples were stored at −70 °C.

### Next generation RNA sequencing analyses (RNA-Seq)

HUVECs (passage 3) were used for the RNA isolation and analyses. Following rRNA depletion, the remaining RNA fraction was used for library construction and subjected to 100 bp paired-end sequencing on an Illumina HiSeq 2000 instrument. Sequencing reads were aligned to the human reference genome assembly (hg19) using TopHat^31^. Transcript assembly and estimation of the relative abundances were carried out with Cufflinks^32^. The resulting data were validated with qRT-PCR.

### Measurement of mRNA and miRNA levels using quantitative Real Time PCR (qRT-PCR)

We used TaqManOne-Step RT-PCR Master MixReagents (Applied Biosystems) as described previously^33^,^34^,^35^ using the manufacturer’s protocol. The relative expressions were calculated using the comparative relative standard curve method^36^. We used *18 S* rRNA as the relative control for our studies. We also validated this relative control against another housekeeping gene, TATA-binding protein (*TBP*). As relative controls for miRNA quantification, we validated and used *RNU44* and *RNU48*. TaqMan probes ids used were: *18 S* - Hs99999901_s1; *TBP* - Hs4332659_m1; *HIF1A* - Hs00153153_m1; *RNU44* - 001094; *RNU48* - 001006; *hsa-miR-429* - 001024, *HIF3A* - Hs00541711_m1; *DDIT4* - Hs01111686_g1. In order to show that miR-429 levels undergo specific changes during hypoxia, we followed the expression of another miR~200 family member miR-141 (hsa -miR-141 - 000463) as shown in Supplemental Figure 5.

### miRNA analogs and target protector transfections

miR-429 mimic (id MC10221) and antagomir (id MH10221) were purchased from Ambion. HUVECs were transfected using the Lipofectamine RNAiMax according to manufacturer’s protocol. miR-429 mimic and antagomir were used at final concentrations of 10 nM and 20 nM, respectively. The transfected cells were cultured for 2 days prior to further analysis. The degree of miRNA over-expression or knockdown was determined by qRT-PCR. Target protectorswere purchased from Qiagen and directed against the *HIF3A* (5′-GTAGAGACGGGGTTTTGCCAAGTTGGAGGGGCTGGTCTTG-3′) and *HIF1A* (5′-ACATAAATAATAATGCTTTGCCAGCAGTAC-3′) mRNAs. Target protectors were used at final concentration of 600 nM. cel-miR-67 was used as a control (Ambion assay id MC22484). The transfected cells were cultured for 2 days prior to further analysis. As an additional control, Ambion siRNA Negative Control 1 no. 4390843 was used as well.

### Western Blots

The procedures were performed as described previously in^10^,^33^. The primary antibodies used were: HIF-1α (Abcam ab16066 (1:150); HIF-3α (Sigma-Aldrich, AV39936 (1:800)) and beta actin (Abcam, AB1801 (1:1000). After the washing steps, the membranes were incubated with goat anti-rabbit IgG (H + L) or with goat anti-mouse IgG (H + L) HRP-conjugated secondary antibodies (BioRad) and detected using ECL (Amresco). Since hypoxia affects a number of proteins commonly used as loading controls, finding the proper housekeeping control protein for normalization is problematic^37^. For example, endothelial cells during hypoxic exposure change their morphology, i.e., tube formation, and this affects both beta actin and tubulin levels^38^,^39^,^40^. Furthermore, HIF1 changes affect glucose metabolism and thus GAPDH levels^37^,^39^ Prolonged hypoxia inhibits global cellular protein translation and transcription rates^41^,^42^. We have tested numerous protein controls for their stability during the time course of hypoxia including beta actin, cofilin, tubulin and GAPDH. Unfortunately, all of them showed variability during the hypoxia time course. Whereas, normalizing the blots to total protein levels allowed us to overcome this limitation. Densitometry was performed using Image Lab software v. 4.1 (BioRad) both prior to the transfer (gel) and after the transfer (membrane). No significant differences in protein distribution were detected between these two methods. The blots shown are representative and the calculations were based on 2 or more independent experiments.

Furthermore, when applicable, all analyzed samples were exposed to hypoxia for the same time period, and we included beta actin-based normalization. The potential direct or indirect effects of miR-429 were eliminated since 3′UTR of ACTB does not have a miR-429 binding sequence, and more importantly, miR-429 mimic and inhibitor did not affect ACTB levels in next generation sequencing experiments.

### Statistical analysis

Results were expressed as means ± standard deviations (SD). Statistical significance among means was determined using the Student’s t-test (two samples, paired and unpaired). Pearson product-moment correlation tests^43^ were performed with SigmaPlot software.

## Additional Information

**How to cite this article**: Janaszak-Jasiecka, A. *et al.* miR-429 regulates the transition between Hypoxia-Inducible Factor (HIF)1A and HIF3A expression in human endothelial cells. *Sci. Rep.* **6**, 22775; doi: 10.1038/srep22775 (2016).

## Supplementary Material

Supplementary Information
