# Supplementary material for: miR-429 regulates the transition between Hypoxia-Inducible Factor (HIF)1A and HIF3A expression in human endothelial cells
Source: Sci Rep. 2016 Mar 8;6:22775. doi: 10.1038/srep22775 (PMC4782134; doi:10.1038/srep22775)
Supplement: Supplementary Information [file srep22775-s1.pdf]

SUPPLEMENTARY INFORMATION

miR-429 regulates the transition between Hypoxia-Inducible Factor (HIF)1A and HIF3A expression in human endothelial cells

Anna Janaszak-Jasiecka, Sylwia Bartoszewska, Kinga Kochan, Arkadiusz Piotrowski; Leszek Kalinowski; Wojciech Kamysz, Renata J. Ochocka, Rafał Bartoszewski, and James F. Collawn

Janaszak & Bartoszewska et al, *Supplemental* Figure 1

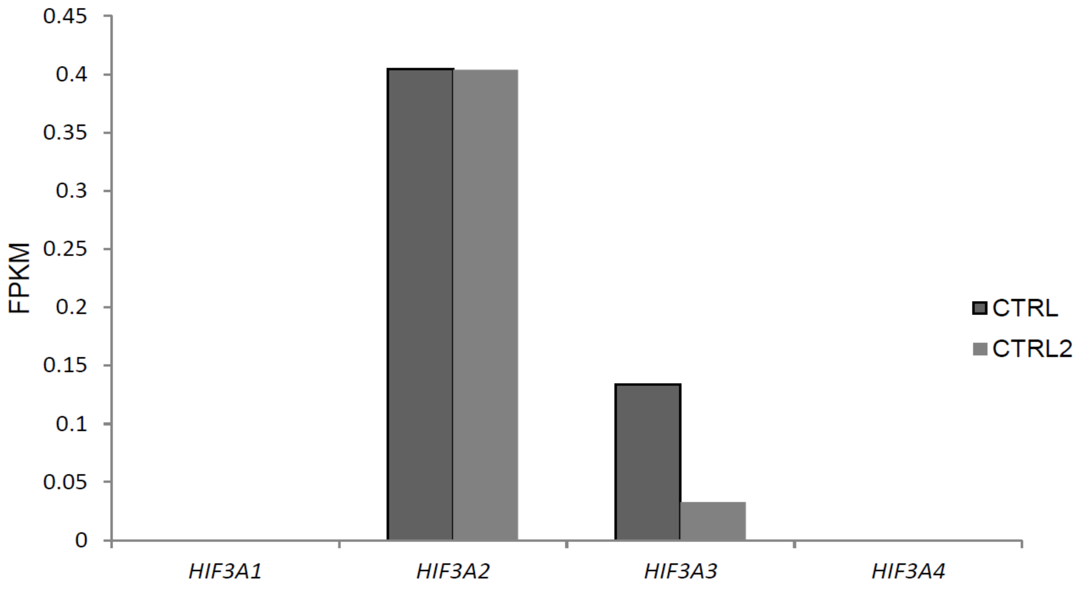

Supplemental Figure 1. The Next Generation Sequencing (NGS) analysis of *HIF3A* isoforms relative expression (Fragments Per Kilobase of transcript per Million mapped reads). In HUVECs, expression of only 2 isoforms was observed in all analyzed samples (two HUVEC control samples, CTRL and CTRL2). Neither the *HIF3A1* (NM_152794.3) nor *HIF3A4* (NM_152796.2) transcript variants were expressed in the samples.

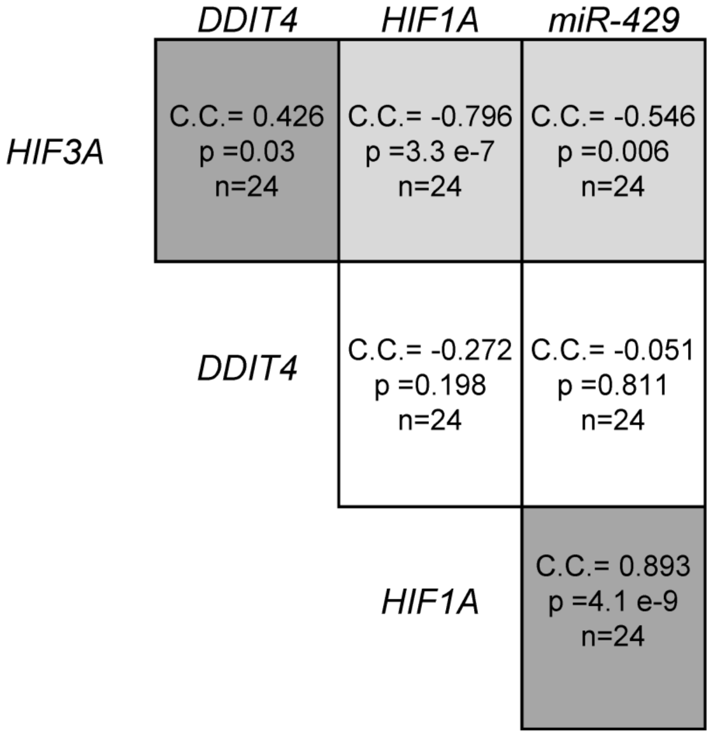

Supplemental Figure 2. Correlation of expression profiles of miR-429, *HIF1A*, *HIF3A2* and *DDIT4*. The qRT-PCR results of hypoxia-induced changes in mRNA and miRNA levels were subjected to Pearson product-moment correlation tests. The pair(s) of variables (mRNA-mRNA and mRNA-miR-429) with positive correlation coefficients (C.C.) and P values below 0.050 increased together (dark grey). For the pairs with negative correlation coefficients and P values below 0.050, one variable decreased while the other increased (light grey). For pairs with P values greater than 0.050 (white), there was no significant relationship between the two variables (n = number of tested values). Pearson product-moment correlation tests were performed with SigmaPlot software.

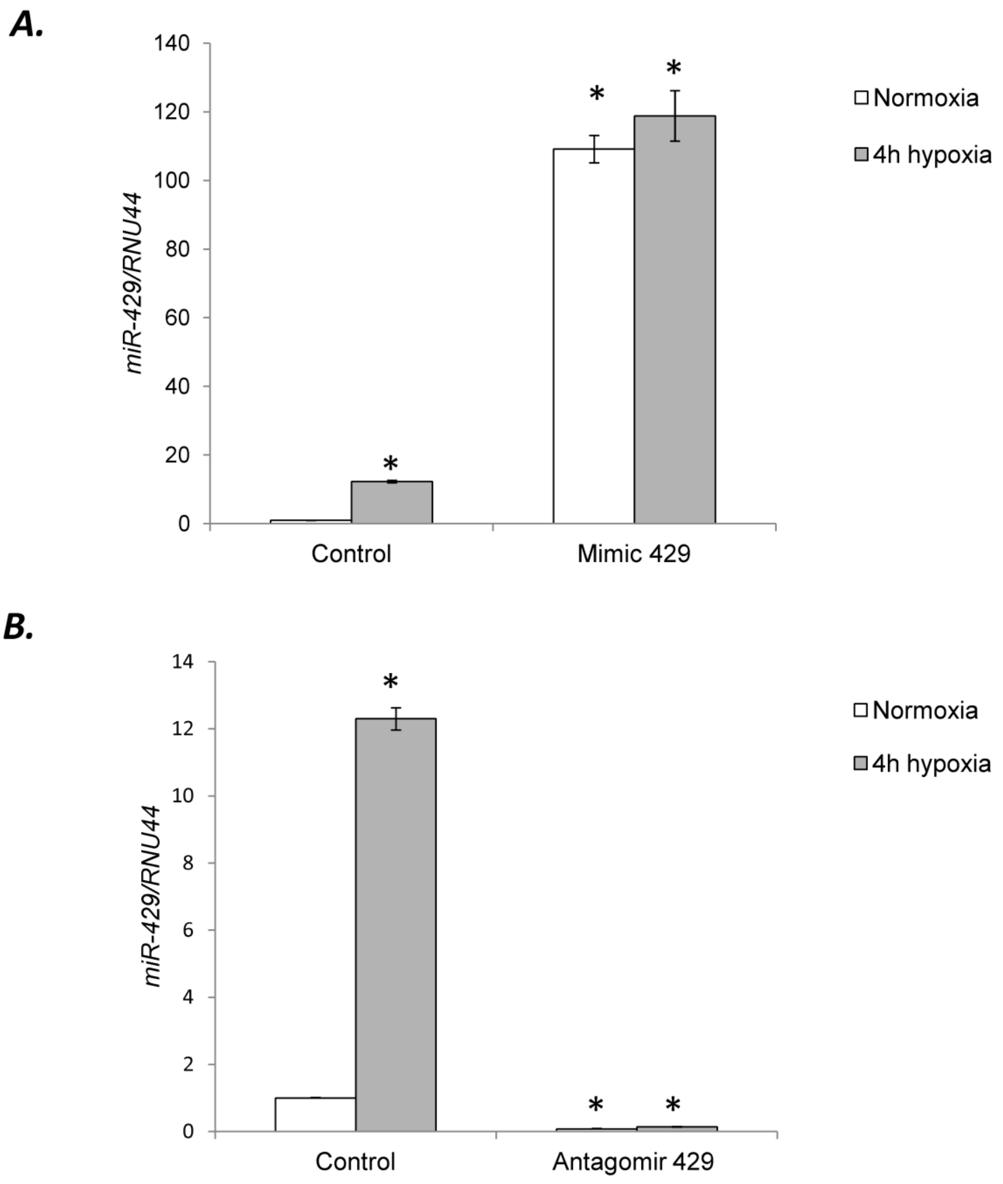

Supplemental Figure 3. Transfections of HUVECs with miR-429 mimic (**A**) and AntagomiR (**B**) were efficient both in normoxia and during hypoxia**.** miRNA levels were monitored in qRT-PCR experiments. The miRNA levels results from 2 independent experiments (n=8) are plotted normalized to RNU44 levels and expressed as a fold change over the control transfection (with c-67). Significant changes ($p<0.05$) are marked with an "*".

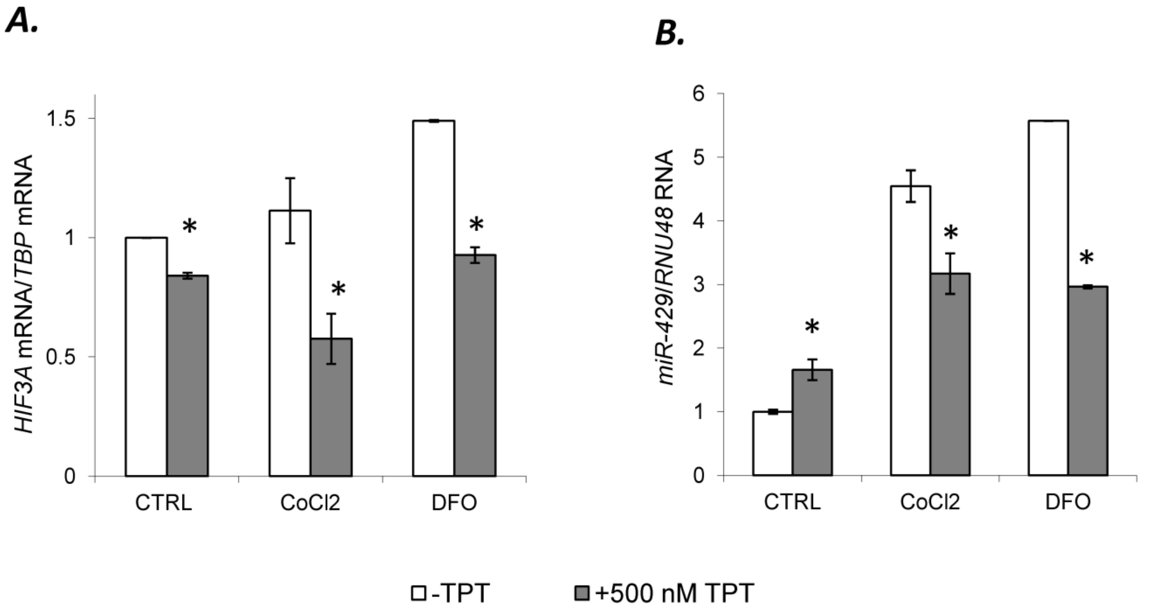

Supplemental Figure 4. HIF-1 induces HIF3A (**A**) and miR-429 (**B**) expression during treatment with hypoxia mimetics. HUVECs were treated with hypoxia mimetics (100 μM desferoxamine (DFO) for 6h or 200 μM $CoCl_2$ for 12h) and with or without (grey and white, respectively) a HIF-1 functional inhibitor (topotecan (500 μM TPT). mRNA and miRNA levels were monitored in qRT-PCR experiments. The miRNA levels results from 2 independent experiments (n=8) are plotted normalized to RNU44 levels and expressed as a fold change over the untreated control. The mRNA levels results from 2 independent experiments (n=12) are normalized to TBP mRNA levels and expressed as a fold-change over the untreated control. Significant changes ($p<0.05$) are marked with an "*"

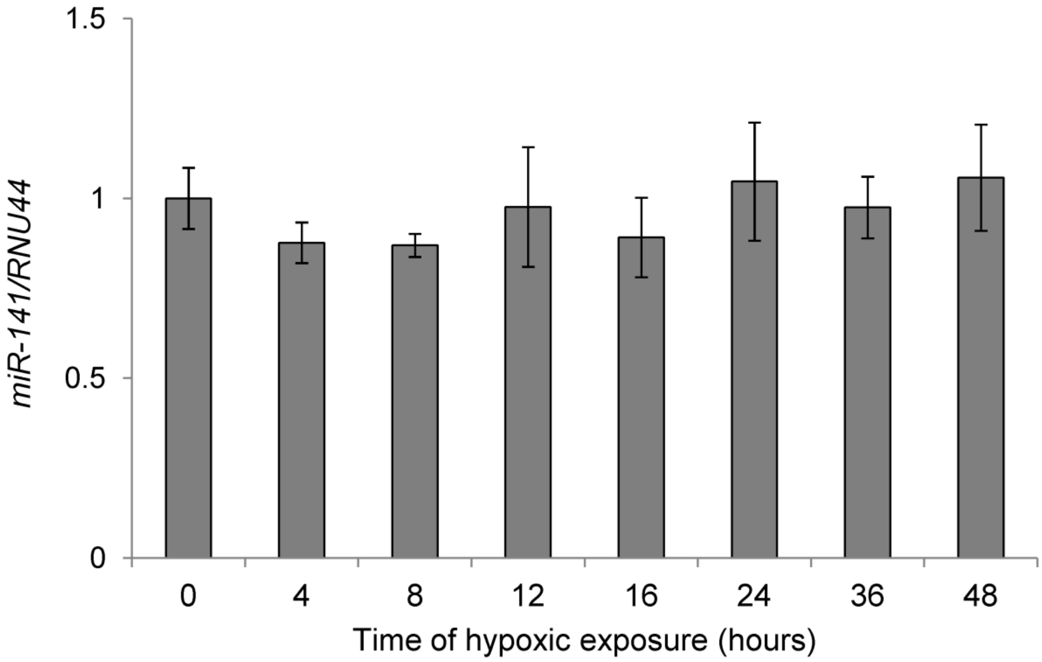

Supplemental Figure 5. Hypoxia does not affect expression profile of miR-141 in HUVECs. The miRNA levels were monitored in quantitative real-time PCR experiments. The results from 2 independent experiments (n = 8) are plotted normalized to *RNU44* and expressed as a fold change over the normoxic control (*$p < 0.05$).
